# STM, SECPM, AFM and Electrochemistry on Single Crystalline Surfaces

**DOI:** 10.3390/ma3084196

**Published:** 2010-08-05

**Authors:** Holger Wolfschmidt, Claudia Baier, Stefan Gsell, Martin Fischer, Matthias Schreck, Ulrich Stimming

**Affiliations:** 1 Department of Physics E19, Technische Universität München, James-Franck-Straße 1, 85748 Garching, Germany; E-Mails: hwolfsch@ph.tum.de (H.W.); cbaier@ph.tum.de (C.B.); 2 Experimentalphysik IV, Institut für Physik, Universität Augsburg, Universitätsstraße 1, 86135 Augsburg, Germany; E-Mails: Matthias.Schreck@physik.uni-augsburg.de (M.S.); stefan.gsell@physik.uni-augsburg.de (S.G.); martin.fischer@physik.uni-augsburg.de (M.F.); 3 Zentrum für Angewandte Energieforschung Bayern e.V. (ZAE Bayern), Walther Meißner Straße 6, 85748 Garching, Germany; 4 Nanotum, Technische Universität München, James-Franck-Straße 1, 85748 Garching, Germany

**Keywords:** STM, SECPM, AFM, single crystalline surfaces, electrochemistry, electrocatalysis

## Abstract

Scanning probe microscopy (SPM) techniques have had a great impact on research fields of surface science and nanotechnology during the last decades. They are used to investigate surfaces with scanning ranges between several 100 μm down to atomic resolution. Depending on experimental conditions, and the interaction forces between probe and sample, different SPM techniques allow mapping of different surface properties. In this work, scanning tunneling microscopy (STM) in air and under electrochemical conditions (EC-STM), atomic force microscopy (AFM) in air and scanning electrochemical potential microscopy (SECPM) under electrochemical conditions, were used to study different single crystalline surfaces in electrochemistry. Especially SECPM offers potentially new insights into the solid-liquid interface by providing the possibility to image the potential distribution of the surface, with a resolution that is comparable to STM. In electrocatalysis, nanostructured catalysts supported on different electrode materials often show behavior different from their bulk electrodes. This was experimentally and theoretically shown for several combinations and recently on Pt on Au(111) towards fuel cell relevant reactions. For these investigations single crystals often provide accurate and well defined reference and support systems. We will show heteroepitaxially grown Ru, Ir and Rh single crystalline surface films and bulk Au single crystals with different orientations under electrochemical conditions. Image studies from all three different SPM methods will be presented and compared to electrochemical data obtained by cyclic voltammetry in acidic media. The quality of the single crystalline supports will be verified by the SPM images and the cyclic voltammograms. Furthermore, an outlook will be presented on how such supports can be used in electrocatalytic studies.

## 1. Introduction

### 1.1. Scanning Tunneling Microscope (STM) in Air and Under Electrochemical Conditions

The invention of the scanning tunneling microscopy (STM) by Binnig *et al.* [1,2] in 1982 paved the way for a variety of scanning probe techniques and the investigation of surfaces on a nanometer scale [3,4,5,6,7,8,9]. Since Hansma and co-workers [10,11] demonstrated that STM can operate in electrolyte solutions, much progress has been made to explore the potential of this technique called electrochemical STM (EC-STM). Especially, the development of a four electrode configuration in the STM in order to carry out complete i*n situ* experiments under potentiostatic control [12,13] has enabled new perspectives for studying electrochemical processes at the solid-liquid interface on a nanometer or even atomic scale. 

The tunneling current that serves as feedback signal in the STM setup is attributed to the quantum mechanical tunneling effect which occurs when the wave functions of a metallic tip and the substrate atoms overlap, *i.e.*, the STM maps the local density of states (LDOS). Thereby, electrons are able to tunnel through a potential barrier consisting of extremely thin, insulating layers such as vacuum, air or liquids with a potential barrier height between 1 eV and 4 eV [14,15]. STM images are recorded while the tip scans in x-y direction over the sample surface and the tunneling current flows perpendicular to the surface. Depending on the operation mode a feedback loop controls the movement of the tip in order to maintain a constant tunneling current (constant current mode) or the tip scans without feedback control, keeping the distance between tip and substrate constant, and measuring the tunneling current (constant height mode). In general STM images show a convolution of electronic properties and the topography of the sample surface. 

### 1.2. Atomic force microscope (AFM)

In 1986 Binnig *et al.* [16] introduced the atomic force microscope (AFM). The AFM probe is a cantilever with a sharp tip normally made of silicon or silicon nitride. While scanning the surface, AFM measures interaction forces between the tip and the sample surface. Since the nature of these forces can be electrostatic, van der Waals, frictional, capillary or magnetic, AFM is also suitable for the characterization of non-conductive surfaces and biological samples. The repulsive and attractive interaction forces, usually in the range between nN and µN, cause the deflection of the cantilever which is typically measured using a laser [17]. Thereby, the laser beam is focused on top of the cantilever and reflected into an array of photodiodes resulting in an electrical signal that is fed into the feedback circuit. When the deflection of the cantilever changes the reflected spot moves on the photodiode and changes the electrical signal. 

AFM imaging modes can be divided into static and dynamic modes. Being operated in the static mode, the AFM tip touches the sample and senses repulsive forces (contact mode). In the dynamic mode, the cantilever oscillates preventing the tip from touching the surface and the AFM tip senses attractive forces (non-contact mode). This mode avoids that the tip or the surface of the sample are damaged which results in a decreased lateral resolution compared to contact mode. The attractive forces result in an amplitude, frequency or phase shift of the tip oscillation, which can be used as feedback control parameter to map the surface [18,19,20]. 

### 1.3. Scanning Electrochemical Potential Microscopy (SECPM)

In 2004 Woo *et al.* [21] reported on a modified EC-STM with a miniaturized potential probe in order to measure the local potential of solid-liquid interfaces with subnanometer spatial resolution. In 2007 this technique, now termed scanning electrochemical potential microscopy (SECPM), was established in the group of Allen J. Bard [22] where STM was also used to a large extent to investigate supports such as Au [23], HOPG [24] and Cu [25].

In order to determine the electrode surface potential in polar liquids or electrolytes, SECPM uses the potential gradient present in the electrochemical double layer (EDL) formed at the solid-liquid interface. The hardware is similar to an EC-STM, the only modification consists of replacing the current pre-amplifier by a high input impedance potential difference amplifier. Since both imaging techniques STM and SECPM are implemented in one head combined EC-STM/SECPM, studies of the same area of an electrode are possible, *i.e.*, potential maps of the surface obtained at constant potential SECPM can be directly compared to the images of the local density of states (LDOS) obtained in constant current mode of the electrochemical STM. 

Recently, it was shown that SECPM can be utilized to investigate the metal content and distribution of tungsten modified diamond-like carbon (DLC) films [26]. Furthermore, SECPM is a promising technique for imaging biological samples such as enzymes and proteins immobilized on electrode surfaces providing a resolution higher than EC-STM [27]. Since no electron transfer between the low conductive biomolecule and the electrode is necessary in the case of SECPM, potential mapping of horseradish peroxidase (HRP) adsorbed on HOPG, allowed identification of the heme group within the protein pocket. 

In addition, SECPM also offers the possibility to map the potential distribution of the interface in x-z direction using the SECPM tip as potential probe [21,22]. However, in order to interpret these potential curves quantitatively, it is necessary to understand how the presence of a probe influences the original EDL at the electrode or *vice versa*. Since the metallic potential probe in contact with the electrolyte also forms an EDL it can interact and overlap with the EDL at the electrode at close distances. The measured interfacial potential results from the overlap of both EDLs. Diffuse double layer interactions between two parallel plates [28], heterogeneously charged colloidal particles [29], or charged particles near surfaces [30], have already been considered in the past as a comprehensive theory for potential profiling. First attempts at an electrostatic approach, directly aimed at the SECPM experiments, using finite element method (FEM) simulation to compute the EDL potential, measured with the metallic probe, were reported by Hamou *et al.* [31,32]. Double layer effects caused by the geometry of the tip apex and the free area of the tip are included in this model. It was found that the shape of the metallic apex affects the ion distribution in the nanogap resulting in an electroneutral region between tip apex and electrode. Consequently, an overall explanation for the SECPM technique cannot easily be given here. Further experimental, as well as theoretical approaches, have to be done to clarify the feasibilities and limitations of this new technique including all above mentioned aspects.

### 1.4. Single Crystals and Single Crystalline Supports

Well defined and oriented surfaces are important requirements in electrochemical surface science to distinguish between the properties of the surface and the investigated electrochemical reaction. These can be absorption and adsorption processes, oxidation or reduction of different molecules, and surface changes such as reconstruction phenomena. Fundamental understanding can be achieved using single crystal surfaces. Polycrystalline electrodes are too complex in geometric and electronic structure and it is almost impossible to relate the influence of the individual surface structure to processes and reactions. There are several ways to prepare single crystals for use in electrochemistry ranging from ultra high vacuum (UHV) preparation, electrochemical treatment, mechanical cutting and polishing, or a combination of all of these [33,34,35,36]. Although, in literature much work was done on single crystals, the preparation of single crystals is time-consuming and complex. 

An alternative route for the preparation of single crystal metal surfaces represents the deposition of thin, heteroepitaxial, films on foreign substrates such as MgO [37] or Al_2_O_3_ [38]. Recently, single crystal metal films of iridium (Ir), rhodium (Rh), platinum (Pt) and ruthenium (Ru) were successfully grown on Si(001) or Si(111) substrates using yttria-stabilized zirconia (YSZ) buffer layers [39]. Ir(001) surfaces provided the base for the first single crystal diamond films on Si(100) [40]. Another approach was followed with Au(111) films deposited on glass supports commercially available from Schroer GmbH. Here, a (111) surface is achieved by annealing of the films, resulting in (111) oriented single crystal grains with random azimuthal orientation. Such high quality single crystalline surfaces can be used for a wide range of investigations with different SPM and standard electrochemical methods. Results from literature describing the behavior of single crystals will be used to determine and evaluate the quality of the surfaces and the purposes for use in electrochemical investigations.

Interesting results were reported by decorating single crystals with thin layers in the range between monolayers down to submonolayers of foreign metals. This often leads to changed chemical and physical behavior of overlayers compared to massive bulk electrodes. Here, important parameters such as coverage of the foreign metal, inter particle distances, particle height and the support are discussed to influence the catalytic activity. Effects caused by the properties of the support material are experimentally [41,42,43,44,45] as well as theoretically [46,47,48,49,50,51] investigated towards catalytic and electrocatalytic properties. The investigations require well defined surfaces such as single crystals to investigate the effects in a precise way. Impressive results were found during the last decade by decorating single crystal surfaces [41,42,43,52,53,54,55,56,57,58]. 

In summary, we present a SPM study in air and electrolyte on single crystalline metal surfaces (Ru, Ir, Rh and Au) and Au(111) single crystals for use in electrochemistry. SPM techniques used are STM, AFM and the novel SECPM technique operating under electrochemical conditions and under air. Since the latter is a new approach to image surfaces, we present results that are compared to AFM and STM. Therefore, we can show that SECPM is a powerful tool to image electrode surfaces down to the nm scale providing new insights into the electrochemical surface properties of different support materials. Besides the imaging technique, standard electrochemical characterization in two different electrolytes with different concentrations was performed. All single crystalline surfaces show the specific features already described in literature with slightly less peak sharpness compared to massive single crystal surfaces. With these supports and the applied imaging techniques, new routes in determining parameters which influence the electrocatalytic activity can be obtained in a high throughput way and compared to recent results from theoretical groups [46,47,48,49,50,51,59,60].

## 2. Results and Discussion 

A comparison of the three techniques, STM, SECPM and AFM, operating in air or under electrochemical conditions in constant current, constant potential and contact mode, is shown in order to evaluate the advantages and disadvantages of the different methods depending on the sample properties. The results will be compared to electrochemical investigations performing cyclic voltammetry, which is very sensitive to the orientation and quality of the single crystal surfaces.

### 2.1. EC-STM, SECPM and AFM Imaging of the Au(111) Crystalline Surfaces

**Figure 1:**
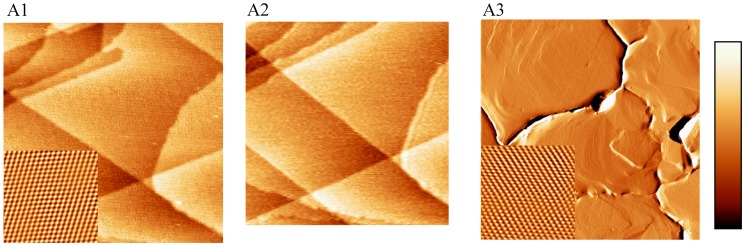
EC-STM, SECPM and AFM of Au(111) single crystalline electrode: (A1) EC-STM (150 nm × 150 nm, h_max_ = 1.5 nm, Inset: 5 nm × 5 nm) U_S_ = 500mV *vs.* NHE, (A2) SECPM (150 nm × 150 nm, h_max_ = 1.5 nm) image of Au(111) single crystal electrode in 0.1 M HClO_4_, U_S_ = 500 mV *vs.* NHE; (A3) Contact Mode AFM in air of a (111) fibre textured gold film (2 µm × 2 µm, U_max _= 0.1V , Inset: 5 nm × 5 nm). Imaging conditions: STM: I_T_ = 1 nA, U_Bias_ = +100 mV, SECPM: ∆U = 5 mV. The insets show atomic resolution.

The typical electrochemical support material single crystalline Au(111) was investigated by EC-STM, SECPM and AFM (Figure 1). Figure 1(A1) shows a typical *in situ* EC-STM image of an Au(111) single crystal in 0.1 M HClO_4_ taken at a potential of 0.5 V *vs.* NHE where no chemical reaction takes place. The image was obtained in constant current mode applying a bias voltage of + 100 mV and a tunneling current of 1 nA. The scanned area of the gold surface shows atomically flat (111) terraces separated by well-defined monoatomically high gold steps. A line scan analysis of the EC-STM image reveals a step height of approximately 2.4 Å which is in good agreement with the theoretical value of 2.35 Å. Furthermore, the angle of the Au(111) terrace edge in the middle of the picture is 58.6 °; 120/60 ° step edges are characteristic of the Au(111) orientation. These results were directly compared with scanning electrochemical potential microscopy (SECPM). The same area was scanned in constant potential mode applying a potential difference of 5 mV between the tip and the Au(111) single crystal electrode. The potential applied to the Au(111) electrode was 0.5 V *vs.* NHE as in the EC-STM experiment. As can be seen in Figure 1(A2), no significant differences between the STM and the SECPM image are observed. The angle of the gold terrace in SECPM is 62.9 ° and the line scan analysis reveals an average step height between 2.4 and 2.5 Å showing the high quality imaging using this SPM technique.

Figure 1(A3) shows a 2 µm × 2 µm overview image of Au(111) obtained in air in contact mode AFM showing grain boundaries of the (111) fibre textured gold film. The image shows the deflection of the cantilever signal. These defect sites were not obtained using a massive Au(111) single crystals in the case of STM and SECPM investigations.

It was possible to resolve the atomic structure of the electrode surface applying STM (Figure 1(A1) Inset) and AFM (Figure 1(A3) Inset). The observed nearest neighbor atomic spacing is about 0.286 nm in STM and 0.276 nm in AFM. Taking the inaccuracy of the techniques into account, both values are in good agreement with the literature value of the fcc lattice constant of 0.289 nm of the Au(111) surface [69]. 

### 2.2. Single Crystalline Surfaces (Ru(0001), Rh(111), Ir(111), Ir(100))

The structure of four single crystalline metal surfaces was investigated via different SPM techniques (EC-STM, SECPM and AFM) and their electrochemical behavior was studied and characterized by performing cyclic voltammetry. 

#### 2.2.1. Ru(0001)

**Figure 2:**
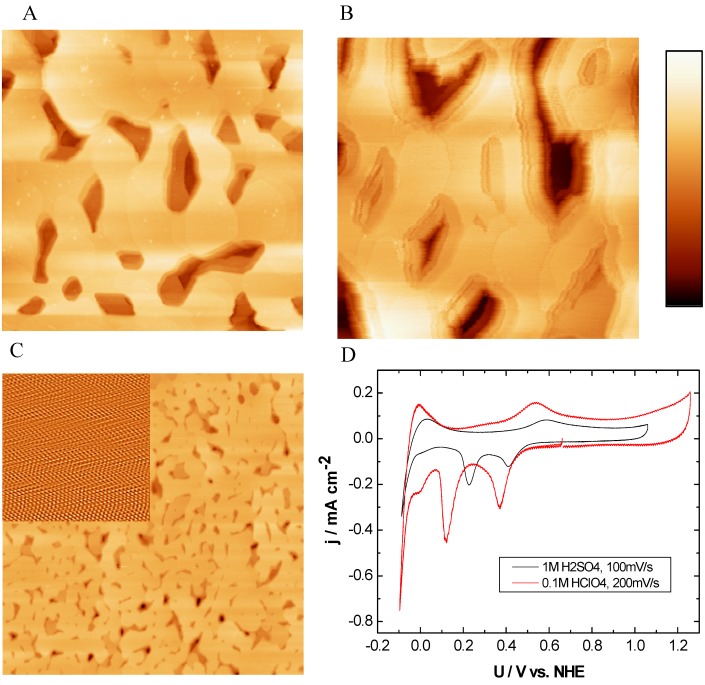
EC-STM, SECPM, AFM and CV of Ru(0001): (A) EC-STM (500 nm × 500 nm, h_max_ = 12.17 nm), U_S_ = 500mV *vs.* NHE, (B) SECPM (500 nm × 500 nm, h_max_ = 17.22 nm) image of Ru(0001) in 0.1 M HClO_4_ at U_S_ = 500 mV *vs.* NHE,** (C)** Contact mode AFM in air (5 µm × 5 µm, h_max_ = 40 nm, Inset: atomic resolution, 12 nm × 12 nm) and **(D)** CVs obtained in 1 M H_2_SO_4_ (black curve) and 0.1 M HClO_4_ (red curve) with a scan rate of 100 mV∙s^-1^ and 200 mVs^-1^, respectively. Imaging conditions: STM: I_T_ = 1 nA, U_bias_ = 100 mV, SECPM: ∆U = 5 mV.

Figure 2 shows the Ru(0001) surface in EC-STM (Figure 2A), SECPM (Figure 2B) and AFM (Figure 2C) down to atomic resolution (Inset Figure 2C). The Ru(0001) structure is characterized by large atomically flat terraces with an average width of up to 100 nm. The terraces were resolved with a similar quality by all three SPM techniques independent of the environmental conditions, *i.e.*, whether recorded in air or in electrolyte. The EC-STM and SECPM images were obtained in 0.1 M HClO_4 _at an electrode potential of 500 mV *vs.* NHE. While the EC-STM image shown in Figure 2A monitors sharp edges of the single crystalline Ru(0001) structure, these edges appear fringed in the SECPM image (Figure 2B). These results indicate that the electronic properties of the step edges, *i.e.*, conductivity mapped by STM and the potential distribution mapped by SECPM, may be different from the electronic properties of the smooth terraces. 

Ruthenium crystallizes in a hexagonal close-packed lattice with a lattice constant of 0.271 nm [70,71]. Applying contact mode AFM, it was possible to resolve the atomic structure shown in the inset in Figure 2C with a measured next neighbor distance of 0.27 nm. This value is in good agreement with the literature data reported above. However, one has to keep in mind that AFM was performed in air at room temperature. Under these conditions oxygen is chemisorbed on the ruthenium surface. LEED experiments and DFT calculations have shown that the O adlayers on Ru(0001) can vary between low coverage up to a full monolayer (Θ = 1) occurring at high gas partial pressure [72,73]. Depending on the coverage, the structure of the O adlayer forms a (2 × 2) or (2 × 1) phase, for high surface coverage, *i.e.*, Θ = 1 the O adatoms sit at the hcp-hollow sites with a (1 × 1) periodicity adopting the lattice constant of the underlying (0001) facet. Assuming that the Ru(0001) single crystalline support is completely covered by oxygen under ambient conditions, it is not clear whether the atomic resolution seen in Figure 2C shows oxygen or ruthenium atoms. However, the observed hcp structure represents the (0001) facet of the ruthenium support. 

Electrochemical characterization on Ru(0001) was done via cyclic voltammetry in sulfuric and perchloric acid in different concentrations. The CVs of Ru(0001) obtained in 1 M H_2_SO_4 _(black curve, Figure 2D) and 0.1 M HClO_4_ (red curve, Figure 2D) were recorded with a scan rate of 100 mVs^-1^ and 200 mVs^-1^ respectively, show well pronounced peaks. The formation of ruthenium hydroxide starts at different potentials depending on the electrolyte and is clearly seen in the anodic peaks at around 0.6 V *vs.* NHE. The formation of RuO_2_ at potentials higher than around 1 V *vs.* NHE was avoided [74]. The two sharp cathodic peaks at 400mV *vs.* NHE are at similar potential, the more negative ones differ by 100 mV. Both cathodic peaks were ascribed to represent the OH reduction and the hydrogen adsorption. Also a specific adsorption of anions is discussed although perchlorate ions are known as weakly adsorbing ions compared to sulfate ions. Sulfate ions are accountable to protect Ru(0001) surfaces from oxidation at lower potentials while supporting a faster reduction process of ruthenium hydroxide [75]. But also the strong dependence of the perchlorate concentration on the hydrogen adsorption was found in our investigations according to [76]. All observed features are also reported on Ru single crystals surfaces in literature [36,75,77,78] showing the high quality of the single crystalline surfaces for electrochemical investigations.

#### 2.2.2. Rh(111)

**Figure 3:**
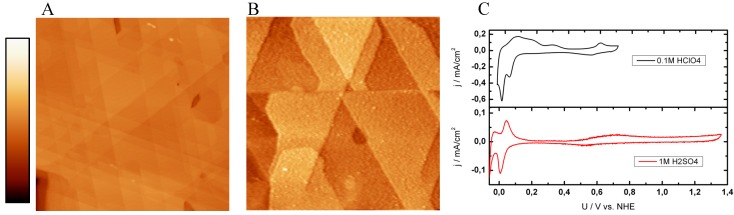
EC-STM, SECPM and CV of Rh(111): (A) *In situ* EC-STM (500 nm × 500 nm, h_max_ = 10 nm) U_S_ = 500mV *vs.* NHE, (B) SECPM (200 nm × 200 nm, h_max_ = 4 nm) and image of Rh(111) in 0.1 M HClO_4_ at U_S_ = 500 mV *vs.* NHE (C) CVs obtained in 0.1 M HClO_4_ (black curve) and 1 M H_2_SO_4_ (red curve) with a scan rate of 100 mVs^-1^. Imaging conditions: STM: I_T_ = 1 nA, U_bias_ = 100 mV, SECPM: ∆U = 5 mV.

Figure 3 shows the EC-SPM and the cyclic voltammetry study of Rh(111). The EC-STM (Figure 3A) and SECPM (Figure 3B) images were obtained in 0.1 M HClO_4_ applying an electrode potential of 500 mV *vs.* NHE. Both images show a closed rhodium layer with a regular pattern of the typical (111) triangular surface structure. The (111) terraces have an average width of 80 nm. Step heights are evaluated by a detailed line scan analysis; they are 0.210 nm for EC-STM (Figure 3A) and 0.222 nm for SECPM (Figure 3B). These values are in good agreement with the Rh(111) monoatomic step height which can be calculated by the lattice constant a of bulk rhodium according to h = 3^-1/2^ a. Taking the theoretical (a = 0.383 nm) [79] and experimental (a = 0.380 nm) [80] fcc lattice constant of rhodium into account, the step height is 0.221 nm and 0.219 nm, respectively. While the EC-STM image reveals the typical 120/60 ° step edges of the (111) structure with an accuracy of ± 1°, the SECPM monitors the Rh(111) structure slightly distorted. The angles are different than 60 °, e.g., α has a value of 62.8 °, whereas β accounts only 53.8 °. These results probably arise from scan artifacts due to the necessary slow scan rate in SECPM mode due to a stronger influence of thermal drift compared to the higher scan rate in STM. 

Rh(111) single crystalline surfaces were investigated in sulfuric and perchloric solutions of different concentrations. For direct comparison the CVs in 1 M H_2_SO_4_ (red curve) and 0.1 M HClO_4_ (black curve) are shown in Figure 3C. Due to the difficulties in Rh single crystal preparation [81] there are only a few reports in literature compared to work on, for example, Pt and Au single crystals. The CV in sulfuric acid (red curve in Figure 3C) has three characteristic regions. The hydrogen adsorption and desorption is well pronounced indicated by the sharp pair of peaks which are observed before the hydrogen evolution. At more positive potentials, the surface is covered with a (√3 × √7) (hydrogen-) sulfate adlayer [82,83] which was resolved by *in situ* electrochemical STM by Wan *et al.* [84] which is similar to the work of Funtikov *et al.* on Pt(111) [85,86]. At potentials more positive than 0.6 V *vs.* NHE, a formation of surface oxides on terraces can be observed due to the broad peak. All specific peaks are comparable to the work of Sung *et al.* [82] and Xu *et al.* [87]. A CV of Rh(111) in perchloric acid is also shown a in Figure 3C (black curve) which is slightly different from that obtained in sulfuric acid (red curve) and peak sharpness is not as clear as observed for massive single crystals [84,88]. The most important difference is the second peak in the hydrogen region. According to Clavilier *et al.* [88] the reduction of perchlorate ions and the adsorption of reduction products takes place at potentials slightly positive from the hydrogen adsorption/desorption or even overlaps. 

#### 2.2.3. Ir(111) and Ir(100)

**Figure 4:**
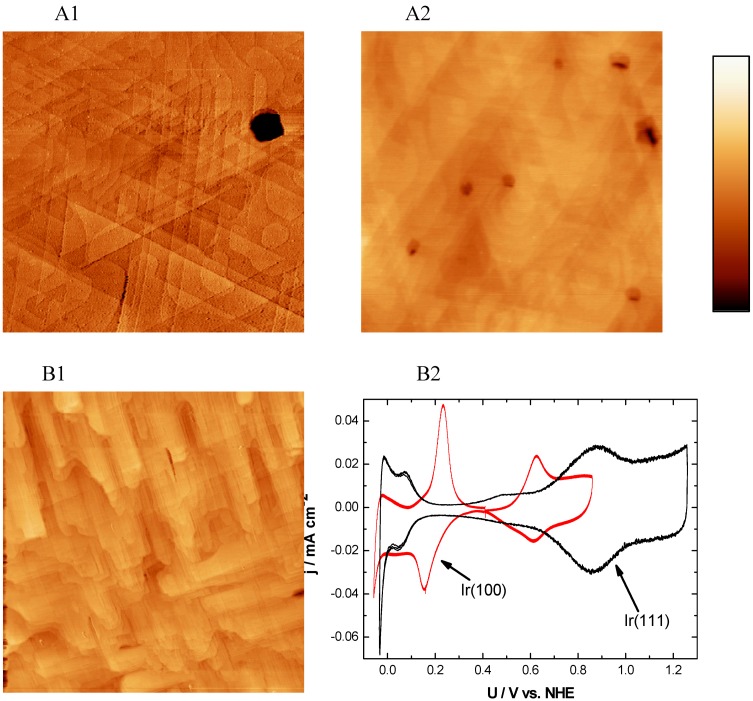
SECPM, AFM and CV of Ir(111) and Ir(100): (A1) SECPM image of Ir(111) (500 nm × 500 nm, h_max_ = 5.5 nm) in 0.1M HClO_4_ at U_S_ = 500 mV *vs.* NHE and (A2) Contact mode AFM image of Ir(111) obtained in air (1000 nm × 1000 nm, h_max_ = 5 nm) image of Ir(111) (B1) STM image of Ir(100) in air (500 nm × 500 nm, h_max_ = nm) and (B2) CVs of Ir(111) and Ir(100) in 1 M HClO_4_ and 0.01 M HClO4 obtained with a scan rate of 100 mVs^-1^ and 50 mVs^-1^ . Imaging conditions: STM: I_T_ = 1 nA, U_bias_ = 100 mV, SECPM: ∆U = 5 mV.

As a neighbor in the periodic system, Ir behaves similar to Pt resulting in related physical and chemical properties. Here we show SPM and electrochemical studies of single crystalline Ir surfaces with (111) and (100) orientation. As can be seen in Figure 4, A1 and A2 typical (111) oriented surfaces were imaged with SECPM and AFM. In a scan area of 500 nm × 500 nm Ir(111) shows well defined triangle structures with extended surfaces. The typical angle of 60 ° was only observed at an imaging scan rate larger than 1 Hz in scan areas smaller than 50 nm × 50 nm. Due to the necessary scan rate, smaller than 0.2 Hz in the SECPM mode in images such as Figure 4(A1), the 60° was not achieved. This can be ascribed to scan artifacts due to thermal drifts causing piezo movements which are more noticeable at slower scan rates. Contact mode AFM images with a higher scan rate result in a higher precision with regard to thermal effects, as can be seen in Figure 4(B2). The holes indicated by the dark brown color in both images show defect sites from the growth process. Evaluating the average step height leads to a value of between 0.24 and 0.25 nm for SECPM measurements. STM images reveal a step height of 0.23 nm which is not shown here. The observed value is in line with literature of 0.221 nm which was calculated from the fcc lattice constant of 0.383 nm [80]). These results indicate the high precision of the different SPM systems. Especially SECPM is able to resolve this in vertical direction as accurately as the STM.

Ir(100) was also investigated with electrochemical and SPM methods. A typical image, obtained by STM in air, is shown in Figure 4(B1). It is clearly seen that the structure is completely different compared to the (111) oriented one. For an overview, a 500 nm × 500 nm picture is shown. The defect density is not as large as for Ir(111) resulting in a smooth surface with a RMS value of 1 nm. Well pronounced 90° angles are visible with terrace widths of up to 50 nm. 

Both surfaces were electrochemically investigated in H_2_SO_4_ as well as HClO_4_ in concentrations ranging from 0.01 M to 1 M. The CV in Figure 4(B2) shows Ir(111) in 1 M HClO_4_ in black and Ir(100) in 0.01 M HClO_4_ in red recorded with a scan rate of 100 mVs^-1^ and 50 mVs^-1^. The hydrogen and the hydroxide adsorption regions are clearly visible for Ir(111) at typical potentials reported also in literature [89,90]. Ir(100) shows also a pronounced CV with all typical peaks reported in [88,89,90,91,92]. The large peaks around 200 mV *vs.* NHE indicate the adsorption and desorption of hydrogen whereas the small peaks around 650 mV *vs.* NHE represent the adsorption and desorption of OH^-^ groups. Comparing this to the CV in [91] the very small prepeaks in the OH^-^ regime could not be observed in the present study. All peaks are not as sharp as they have been observed for perfect single crystals. The broadening of the peaks is an indicator for a smaller terrace width and a higher defect density compared to single crystals. Nevertheless, the electrochemical data are very sensitive to structural properties and still clearly show the characteristic properties of the Ir(111) and Ir(100) surfaces.

## 3. Experimental Section 

### 3.1. Materials and Instrumentation

The Au(111) single crystal electrode (MaTeck) was 10 mm in diameter and 1 mm thick, Au(111) films on mica were obtained from arrandee (Schroer GmbH) consisting of 0.7 mm borosilicate glass, 2.5 nm chromium and 250 nm gold layer with a square size of 11 mm × 11 mm. 

The electrolytes were prepared from perchloric acid (HClO_4_, 70%, Merck, suprapure), and sulfuric acid (H_2_SO_4_, 95–98%, Merck, suprapure) with ultraclean water obtained from a Millipore-Milli-Q system (18.2 MΩ cm, 3 ppm total organic carbon). 

Peroxymonosulfuric acid (Caro’s acid) was prepared with H_2_SO_4_ (95–98%, Merck, p.a.) and H_2_O_2 _(33%, Merck, p.a.) at a volume ratio of 1:1 for cleaning glassware, all Teflon parts and noble metals. 

HCl (32%, Merck, analytical grade) was used as etching solution for the STM and SECPM tips made up of gold or palladium wire. Nobel metal wires had a diameter of 0.25 mm and were purchased from Carl Schäfer GmbH Co.KG (Germany). 

Argon gas (4.8) from Linde AG (Germany) was used in order to flush the electrolyte and to remove dissolved oxygen from the experimental setup. 

STM, EC-STM, SECPM and AFM measurements were performed using an electrochemical Veeco Multimode system with the Veeco universal bipotentiostat, a combined STM/SECPM head or an AFM head, a Nanoscope 3D Controller and the Nanoscope 5.31r2 software. All experiments were performed using the E scanner. 

For imaging in air the electrodes were mounted on a paramagnetic disc using double-sided adhesive tape (CMC Klebetechnik, Germany). All i*n situ* experiments were performed in small PTFE cells with an electrolyte volume of 100–200 µL. In order to remove organic contamination, the PTFE cell was cleaned with Caro’s acid and then thoroughly rinsed with Millipore water before each experiment.

When not otherwise stated the STM experiments were performed by applying a bias voltage of U_Bias_ = U_Tip_ − U_S_ = +100 mV and a tunneling current I_T_ = 1 nA. SECPM images were recorded with a potential difference setpoint of ∆U = U_S_ – U_Tip_ = 5 mV.

EC-STM/SECPM tips were prepared by electrochemical etching of a 0.25 mm gold or palladium wire in HCl (32%) with a voltage of 1.65 V dc. In order to reduce Faradaic leakage currents during the EC-STM and SECPM measurements, the tips were insulated with Apiezon wax (Plano, Germany). 

In the EC-STM/SECPM cell gold/gold oxide and pure gold wire electrodes were used as reference electrode and counter electrodes, respectively. Before and after each experiment the potential of the gold-gold oxide reference electrode was measured for stability: Since all experiments were performed in 0.1 M HClO_4_ the gold/gold oxide electrode was measured *versus* a mercury/mercury sulfate electrode (Schott, 3610) stored 0.1 M H_2_SO_4_ (660 mV *vs.* NHE) in 0.1 M HClO_4_.

All electrode surfaces were imaged in constant current mode in STM in air and in *in situ* EC-STM, in constant potential mode in SECPM, in contact and tapping mode AFM in air. The STM images were recorded with a tunneling current I_T_ of typically 1nA. The potential of the substrate U_S_ is given with respect to NHE in each figure caption. The bias voltage is determined as U_bias_ = U_tip_ – U_S_ under electrochemical conditions, otherwise the voltage is applied between sample and tip in air mode. The potential setpoint between tip and substrate ∆U for the SECPM mode is also given in the figure caption. The imaging velocity was typically between 0.1 and 1Hz depending on the scanning mode and the scanned area. 

AFM cantilevers were purchased from Veeco. For contact mode triangular cantilevers (NP-S10) and for tapping mode rectangular cantilevers (RTESP) with a resonance frequency of 320 kHz were used.

Image processing was performed with the image software WSxM 4.0 [93]. Raw data of the atomic resolution were processed applying a 2D FFT filter. 

Cyclic voltammetry experiments were performed in diluted HClO_4_ and H_2_SO_4_ electrolytes in a standard three electrode arrangement in an electrochemical glass cell applying scan rates of 100 mVs^-1^ or 200 mVs^-1^ using an Autolab PGSTAT 30 (Metrohm Autolab B.V., Netherlands) with the data acquisition software GPES. 

Ru(0001), Rh(111), Ir(111) and Ir(100) single crystalline surfaces are prepared according to the procedure described in [94] in the group of M. Schreck at the University of Augsburg. Therefore 40 nm thick YSZ films on Si(111) or Si(100) were used as support. The metal films were deposited via e-beam evaporation with a typical thickness of about 150 nm with different growth rates. Details regarding apparatus details, temperatures and growth parameters can be found in [39,94]. The samples which are grown on 4 inch wafers were carved into approx. 1 cm × 1 cm square samples for further use in electrochemical as well as SPM investigations. 

### 3.2. Sample Preparation 

#### 3.2.1. Au(111) electrodes

Before each experiment the Au(111) single crystal was oxidized in 0.1 M HClO_4_ at 1.8 V *vs.* normal hydrogen electrode (NHE) for 2–3 minutes. After removing the formed gold oxides by dipping the electrode into HCl, the electrode was thoroughly rinsed with Millipore water. The Au(111) films on mica were rinsed only with ultrapure water before use. Then the Au(111) single crystal electrode and also the Au(111) film substrates were annealed for several minutes in a Bunsen burner flame until glowing red. After cooling down the crystal in an argon stream for several minutes, the electrode was immediately transferred to the PTFE cell with a droplet of ultrapure water protecting the surface from contamination. Cyclic voltammograms in sulfuric acid served as an electrochemical indicator for the quality of the Au(111) single crystals.

#### 3.2.2. Ru(0001), Rh(111), Ir(111), Ir(100)

The single crystal film electrodes were cleaned in a three step process. After rinsing with ultrapure water the samples were adhered between Teflon tapes with an exposed area of 0.2 cm^2^. Cleaning in ultrasonic bath for 5 minutes in acetone, isopropanol and ultrapure water leads to clean surfaces. Rinsing with ultrapure water before use in electrochemical as well as SPM investigations is the last cleaning step. 

## 4. Conclusions 

Single crystalline surfaces were investigated with three SPM techniques: STM, SECPM and AFM. In addition, cyclic voltammetry was used to investigate the quality and suitability of these surfaces for investigations in electrochemistry. We showed that the SECPM technique based on the potential difference between two electrodes in electrolytes has a resolution which is comparable to STM in air and under electrochemical conditions on the investigated surfaces. 

All presented heteroepitaxially grown Ru, Rh and Ir single crystalline surfaces show high quality. This resulted from the SPM and electrochemical investigations and was discussed with respect to literature on bulk single crystal surfaces. Although some defects due to preparation were found on the surfaces, the quality represents a high standard. This makes them suitable for a wide range of investigations under electrochemical conditions. Especially, the use as support material in electrocatalysis where foreign metals may be deposited onto the substrates will clarify the influence of the support. Large single crystalline regions, single-use electrodes and production technique on 4 inch wafers which yield a large number of identical samples are additional advantages.

We showed that the new developed SECPM and well established STM and AFM offer comparable resolution although the physical background is quite different. In combination with electrochemical investigations single crystalline surfaces were characterized to be excellent supports for further investigations towards substrate effects in electrochemistry. 
